# Digital detector PET/CT increases Centiloid measures of amyloid in Alzheimer's disease: A head-to-head comparison of cameras

**DOI:** 10.1177/13872877241313063

**Published:** 2025-01-26

**Authors:** Ashley Gillman, Pierrick Bourgeat, Timothy Cox, Victor L Villemagne, Jurgen Fripp, Kun Huang, Rob Williams, Rosita Shishegar, Graeme O’Keefe, Shenpeng Li, Natasha Krishnadas, Azadeh Feizpour, Svetlana Bozinovski, Christopher C Rowe, Vincent Doré

**Affiliations:** 1 Health and Biosecurity, Commonwealth Scientific and Industrial Research Organisation, Brisbane, QLD, Australia; 2 Department of Psychiatry, University of Pittsburgh, Pittsburgh, PA, USA; 3 Department of Molecular Imaging & Therapy, Austin Health, Melbourne, VIC, Australia; 4 Melbourne Brain Centre Imaging Unit, The University of Melbourne, Melbourne, VIC, Australia; 5 The Florey Institute of Neuroscience and Mental Health, The University of Melbourne, Parkville, VIC, Australia

**Keywords:** Alzheimer's disease, amyloid, CapAIBL‌, Centiloid, harmonization, neuroimaging, positron emission tomography, quantification, resolution‌, SPM

## Abstract

**Background:**

The introduction of therapeutics for Alzheimer's disease has led to increased interest in precisely quantifying amyloid-β (Aβ) burden for diagnosis, treatment monitoring, and further clinical research. Recent positron emission tomography (PET) hardware innovations including digital detectors have led to superior resolution and sensitivity, improving quantitative accuracy. However, the effect of PET scanner on Centiloid remains relatively unexplored and is assumed to be minimized by harmonizing PET resolutions.

**Objective:**

To quantify the differences in Centiloid between scanners in a paired cohort.

**Methods:**

36 participants from the Australian Imaging, Biomarker and Lifestyle study (AIBL) cohort were scanned within a year on two scanners. Each participant underwent ^18^F-NAV4694 imaging on two of the three scanners investigated, the Siemens Vision, the Siemens mCT and the Philips Gemini. We compared Aβ Centiloid quantification between scanners and assessed the effectiveness of post-reconstruction PET resolution harmonization. We further compared the scanner differences in target sub-regions and with different reference regions to assess spatial variability.

**Results:**

Centiloid from the Vision camera was found to be significantly higher compared to the Gemini and mCT; the difference was greater at high-Centiloid levels. Post-reconstruction resolution harmonization only accounted for and corrected ∼20% of the Centiloid (CL) difference between scanners. We further demonstrated that residual differences have effects that vary spatially between different subregions of the Centiloid mask.

**Conclusions:**

We have demonstrated that the type of PET scanner that a participant is scanned on affects Centiloid quantification, even when scanner resolution is harmonized. We conclude by highlighting the need for further investigation into harmonization techniques that consider scanner differences.

## Introduction

The recent approvals of monoclonal antibody therapeutics marks the clinical adoption of the first disease-modifying therapies of Alzheimer's disease (AD) and are causing rapid changes in AD care.^1^ Clinical positron emission tomography (PET) imaging and quantification of amyloid-β (Aβ) levels is increasingly important: expert panels recommend the clinical use of Aβ imaging to confirm the presence of cortical Aβ prior to treatment,^2–4^ and adaptive treatment regimens that reduce dose after Aβ reduction indicate future need for follow-up tracking during the treatment course.^5^ In addition, the novel nature of these diagnostics are leading to an increased interest in research trials measuring relatively small differences in Aβ burden. Altogether, robust and accurate quantification methods for Aβ burden quantification in research and the clinic are now of critical importance.

The Centiloid (CL) scale^6^ was introduced to promote consistent Aβ burden quantification across different A β PET tracers and quantification pipelines. The Centiloid scale is anchored using a ^11^C-Pittsburgh Compound B (^11^C-PiB) cohort of young, healthy controls, set to 0 CL, and of mild AD patients, set to 100 CL. The project also introduced a standard processing pipeline along with a neocortical mask which defines the regions of typical amyloid cortical uptake and associated reference regions masks. A calibration method, (termed “level-2 calibration”) was introduced, and has been used to calibrate other tracers^7–10^ and quantification pipelines^9,11–13^ to the Centiloid scale.

A recent review by Bollack et al.^14^ systematically categorized factors affecting Aβ quantification. The Centiloid framework is designed to harmonize some of the effects they highlight, including distribution and affinity differences between tracers and use of different image processing pipelines, but was not intended to control for many extrinsic factors identified. These include: scanner sensitivity (especially with the introduction of long-axial-field-of-view scanners,^15^ rapid changes in time-of-flight resolution^16^), intrinsic resolution, and algorithmic changes during reconstruction. The combined effects of changes of scanner hardware and the manufacturers reconstruction software have been demonstrated to introduce bias in PET quantitative imaging.^13,17–20^ Further, the anchor cohort that the Centiloid scale is derived from includes images from 5 different scanners, raising questions around how, if the framework was extended to harmonize other scanners, it would be anchored without compatibility issues with the original scale. In the majority of studies (including ADNI,^21^ OASIS-3^22^ and AIBL^11^), scanner effects are attributed to only resolution effects, and post-reconstruction resolution harmonization^17^ is used.

While many head-to-head assessments of quantification accuracy between tracers^7–10,23^ and quantification methods^9,24,25^ have been presented, the effect of scanner is unexplored. Previous cross-sectional and longitudinal non-paired harmonization analyses have considered^26^ and identified^27^ scanner effects, but have not reported their magnitude. This work introduces the first in vivo, paired measurement of the scanner effect on Aβ quantification. In this work, we aim to quantify the effect of scanner hardware and associated reconstruction changes on quantification of Centiloid. Further, we show that with recent advances in PET technology, PET scanner difference cannot be accounted for with purely resolution (in terms of point-spread function) effects. We show this both by applying post-reconstruction smoothing and by investigating spatial differences. We present this analysis on a paired sub-cohort of patients within the Australian Imaging, Biomarker and Lifestyle study (AIBL)^28,29^ scanned on two of three investigated PET scanners.

## Methods

### Dataset

A subset of 36 participants of the AIBL study^28,29^ (Table 1) underwent consecutive ^18^F-NAV4694 scanning on two of the three scanners considered in this study, the Siemens Biograph 128 Vision 600 Edge (herein, Vision), the Philips Gemini 64 ToF (herein, Gemini) and the Siemens Biograph 128 mCT (herein, mCT). Participants were scanned between March 2021 and January 2023 according to the most recent protocol published by Fowler et al.,^29^ with time between scans of 56-364 days (mean = 244 days). Acquisitions were reconstructed according to the Standardized Centralized Alzheimer's & Related Dementias Neuroimaging (SCAN) protocol,^30^ with standard corrections including normalization, decay, randoms, scatter and deadtime, and all attenuation corrections were performed using low dose computed tomography. Other parameters on each scanner are given in Table 2. Participants underwent MRI scanning to acquire a 3D magnetization-prepared rapid acquisition gradient echo (MPRAGE) image on either a Siemens Prisma or Siemens Skyra, and the remaining participant was excluded from the standard SPM8 Centiloid analysis but was included in the CapAIBL analysis.

**Table 1.** Number and basic demographics of recruited participants.

	Vision∼Gemini	Vision∼mCT	Gemini∼mCT
N	21	7	8
Ratio female	48%	29%	50%
Age	77.7 [69–92]	80.1 [74–86]	76.7 [65–82]
N (CU/MCI/AD)	16/3/2	4/2/1	7/1/0
MMSE	27.9 [22–30]	27.9 [25–30]	28.6 [26–30]
Days between scan	195 [56–362]	328 [282–364]	290 [216–364]

CU: cognitively unimpaired; MCI: mild cognitive impairment; AD: Alzheimer's disease; MMSE: Mini-Mental State Examination results. Distribution values are given as mean [min – max].

**Table 2.** Acquisition parameters for each scanner.

	Vision	Gemini	mCT
Activity	∼100 MBq (22 participants) ∼190 MBq (6 participants)	∼190 MBq	∼190 MBq
Reconstruction	OSEM 8 iterations 5 subsets	LOR-RAMLA	OSEM 4 iterations 24 subsets
Post filter	None	“SHARP”	None
Time of Flight	On	On	Off

OSEM: ordered subsets expectation maximization; LOR-RAMLA: line-of-response row-action maximum likelihood algorithm; FWHM: full-width at half-maximum; LDCT: low dose computed tomography.

The AIBL study, including the follow-up protocol and subsequent amendments and revisions to the protocol, was approved by the institutional human research ethics committees of Austin Health, St Vincent's Health, Hollywood Private Hospital and Edith Cowan University. All volunteers gave written informed consent before participating in study assessments, and the study was conducted in accordance with the Helsinki Declaration of 1975.

### Centiloid quantification

Centiloid quantification was performed both with the original Centiloid pipeline defined by Klunk et al.^6^ using Statistical Parametric Mapping 8 (SPM8, MATLAB R2019a), and with the CapAIBL PET-only pipeline.^31,32^ The latter are presented in Supplementary Material 3. Briefly, in the SPM8 processing, each scan's MRI was registered to the MNI-152 template using the SPM8 unified segmentation method. Each subject's PET was registered to their corresponding MRI and spatially normalized to the MNI template by propagating the deformation parameters from the MRI-MNI registration. The standardized uptake value ratio (SUVr) was calculated as the ratio of mean activity in the Centiloid neocortical mask^6^ to that in the chosen reference mask, which unless otherwise specified was the whole cerebellum.

To investigate residual spatial effects, we also compare the Centiloid differences for subregions over the cortex and the effect of varying the reference region between commonly chosen regions. We conducted a subregional analysis to investigate the scanner effect on subsets of the Centiloid mask and explore the spatial dependance of Centiloid changes. Subregions were defined by taking the intersection of the Centiloid mask with Automated Anatomical Labeling (AAL) atlas regions.^33^ Additionally, four separate reference regions were compared: whole cerebellum, cerebral gray matter, pons and whole cerebellum in union with white matter.^34^ ^18^F-NAV4694 Centiloid level-2 calibration^6^ was previously reported^11^ and performed on an independent dataset from the cohort used in this study. Level-2 calibration was performed separately for SPM8 and CapAIBL,^31^ and for each reference region.

### Correction for scan delay

Time between scans was limited to less than 365 days to reduce any impact of Aβ accumulation. To correct any residual effects, population disease trajectory curves were used to calculate and correct for expected change in Centiloid between scans.^35–37^ Details are included in Supplemental Material 1.

### Post-reconstruction resolution harmonization

Post-reconstruction resolution harmonization was performed by smoothing images to an equivalent resolution, following the approach of Joshi et al.^17^ Each scanner's resolution was estimated by co-registering a digital Hoffman phantom to a scan of a physical Hoffman phantom, and fitting axial and transaxial smoothing parameters and a scaling factor of the digital phantom to minimize the L2 norm of the difference between the digital and physical images.

$${\mathrm{argmin}}_{\sigma_{xy},\sigma_{z},a}\|\;u-av*k(\sigma_{xy},\;\sigma_{xy},\;\sigma_{z}){\|}^{2}$$

where *u* is the physical phantom image, *v* is the digital phantom image, *σ_xy_* and *σ_z_* are the transaxial and axial measured resolution, *a* is a scale factor, k(x,y,z) is a function producing a Gaussian kernel with a given *x*, *y* and *z* axis standard deviation, and * is the convolution operator.

The appropriate smoothing factors are then $\phi^{2}=\tau^{2}-\sigma^{2}$ , where σ is the measured resolution, τ is the desired equivalent resolution and ϕ is the corrective resolution. Finally, the high-frequency, harmonized image, *w* with ϕ equivalent spatial resolution is given by:

$$w=u*k(\phi_{xy},\phi_{xy},\phi_{z})$$

Resolution harmonization was applied to reach a desired equivalent smoothing, τ , of 6 mm full-width-at-half-maximum (FWHM). To investigate how much smoothing would be required to minimize the differences between the Vision and the Gemini, we also present an analysis of decreasing the harmonized equivalent resolution of the Vision in Supplemental Material 1, contrasting τ= 6 mm, 8 mm, 10 mm and 12 mm to find a value which minimizes the difference in Centiloid between the Vision and Gemini.

### Analyses

Two-sided Wilcoxon signed rank tests were used to directly compare the Centiloid measured on each scanner, as the differences were not Gaussian distributed. All statistical significances are defined by p<0.05 , except where Bonferroni corrections are noted.

Regression analyses were performed to compare each scanner pair at native resolution, and with high-frequency harmonization. Total least squares (TLS) regression was used such that the regression is unbiased to the selection of any reference scanner. In Total least squares regression, the error is measured orthogonal to the regression line from each point, in contrast to ordinary least squares regression, where the error is measured in the dependent variable, meaning the regression is independent of any choice of “reference” scanner. An empirical, one-sided p -value was determined for each linear fit parameter (intercept and slope) as the portion of parameter estimates more extreme than the difference from 0 for intercept and 1 for slope. Additionally, Bland Altman plots with bootstrap 95% intervals are presented in Supplemental Material 1, and the two measurements were considered equivalent if the interval completely contained the horizontal origin (i.e. the bootstrap interval is consistent with a scanner quantification difference of 0 over all Centiloid ranges). In the subregional analyses, the bootstrap distributions of slope parameters between each pair of regions were compared with a two-sided Mann-Whitney U rank test with a Bonferroni correction for the number of tests presented in the analysis.

The participant recruitment on the mCT scanner was low, especially for high CL participants, meaning it was difficult to draw conclusions about the relationships with this scanner. We use an unbiased imputation method to propagate information from other scanner pairs. The description and validation of the method are presented in Supplemental Material 2. The regression analysis was repeated on the imputed data and presented in Supplemental Material 2.

## Results

**Figure 1.**
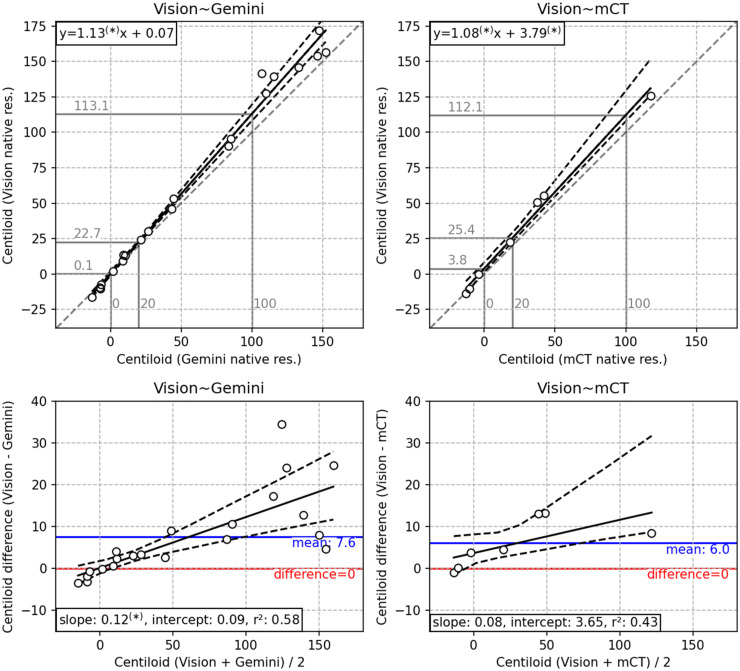
(Top) Scatter plot of each scanner pair, with a total least squares (TLS) regression line (solid), bootstrap 95% confidence interval (dashed), equation (upper left) and the unity mapping (gray, dashed). Mappings are shown for 0, 20 and 100 Centiloid on the x-axis (gray). (Bottom) Bland-Altman plot of each scanner pair with a regression line (solid), bootstrap 95% confidence interval (dashed), mean difference and unity mapping . Asterisks after number indicate significant difference from unity (slope in top row) or zero (intercept in top row, or slope or intercept in bottom row).

**Figure 2.**
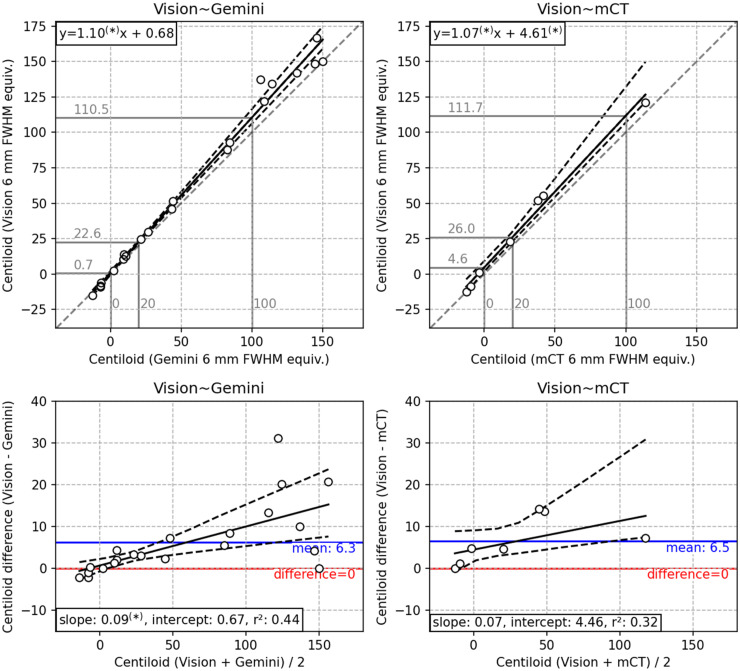
Per figure 1, with 6 mm post-reconstruction resolution harmonization applied.

The results of the statistical analysis are given in Table 3, and the results of the regression analysis are outlined in Table 3 and depicted in Figures 1 and 2. In the regression analysis at native resolution, the Vision had a slope significantly greater than 1 compared against both Gemini and mCT but a small intercept (Table 3), such that scanner differences were seen in participants with high Centiloid (Figure 1). For the Vision∼Gemini scanner pair, the “difference = 0” line lies outside the bootstrap 95% confidence interval (CI) for mean CL > 10 (Figure 1, bottom), meaning there is a discernible scanner difference when CL is greater than 10, but little difference for subjects with no Aβ deposition. For the Vision∼mCT pair, there was again a positive trend, such that the difference = 0 is outside the bootstrap 95% CI for mean CL > −10 (Figure 1, bottom), so although the scanner difference was lesser at low Aβ levels, there is a measurable difference regardless of the level. Due to low sampling of patients with CL > 20 on the Gemini∼mCT scanners, this pair is omitted from analysis, although included in Supplemental Material 2. An image difference comparison within the brain (Supplemental Figure 8) showed that image-based differences in SUVr images are similar to the Centiloid difference comparison.

**Table 3.** Results of statistical and regression analyses between scanner pairs without and with resolution harmonization.

Resolution Harmonization	Scanner Pair	Mean Difference (CL)	Slope (CL/CL)	Intercept (CL)
Native Res.	Vision∼Gemini	+7.6*	1.13*	0.07
	Vision∼mCT	+6*	1.08*	3.79*
6 mm	Vision∼Gemini	+6.3*	1.10*	0.68
	Vision∼mCT	+6.5*	1.07*	4.61*

Mean difference is the first scanner of the pair minus the second. Asterisks indicate significance p < 0.05: for mean difference, using a two-sided Wilcoxon signed rank test with a test value of 0; and for slope and intercept, using an empirical one-sided p-value, that is the number of bootstrap parameter estimates beyond 1 for slope and 0 for intercept.

The same analysis performed with imputation, presented in Supplemental Material 2, yielded consistent results for the Centiloid difference between Vision∼Gemini (Supplemental Figure 9), that the difference is near to 0 at 0 CL, and increases to about +10 CL at 100 CL. The imputation analysis suggests that the difference between the Vision∼mCT scanners is around +3 CL at 0 CL and +13 CL at 100 CL. The imputation analysis also suggests that the Gemini∼mCT scanners are far more consistent, with a constant approximately +1 CL difference that was only significant at lower A β deposition.

With the PET-only Centiloid quantification pipeline, CapAIBL (Supplemental Material 3), differences in Centiloid are increased. All scanner pairs had a larger absolute difference measured with CapAIBL (Supplemental Figure 1^1^) compared with SPM8 (Figure 1), and the slope of error proportional to Centiloid measured for the Vision∼Gemini and Vision∼mCT scanners increased from 1.13 and 1.08 (Figure 1), to 1.18 and 1.10 (Supplemental Figure 1^1^), respectively.

**Figure 3.**
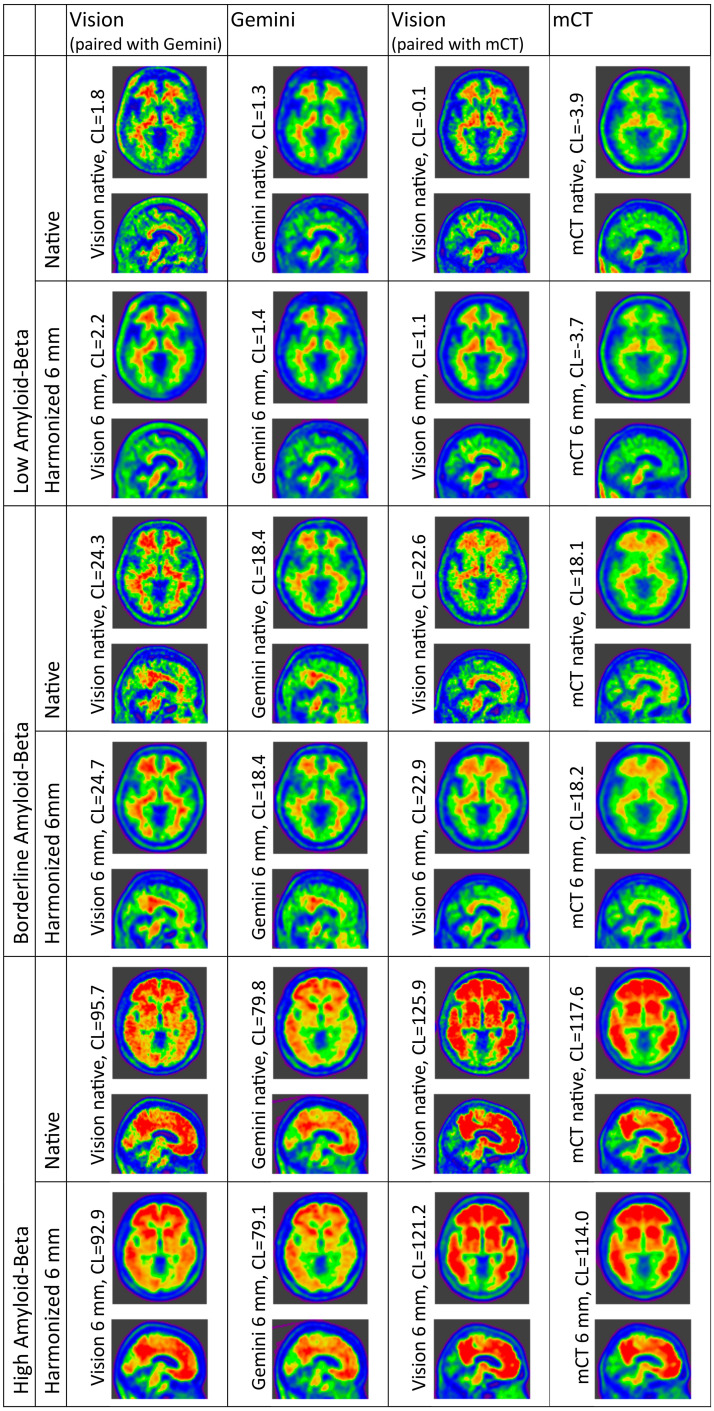
Representative patient scans acquired on each scanner, with and without resolution harmonization applied. Three patients per scanner-pair are depicted, one each with low, borderline and high A β burden. The first and second columns, as well as third and fourth columns, depict the same participant.

Comparison of Centiloid after resolution harmonization to 6 mm FWHM equivalent resolution is displayed in Figure 2, and a representative sample of both native and 6 mm harmonized resolution participants are depicted in Figure 3. Resolution harmonization resulted in a small reduction of the difference between Centiloid from the Vision∼Gemini pairing (mean +6.3 CL compared with +7.6 CL in Table 3) but had little impact on the difference to the trend (Figure 1 compared with Figure 2). Resolution harmonization reduced the Centiloid difference at 100 CL from +13.1 to +10.5 CL (a 20% reduction) but had little effect on the Vision∼Gemini Centiloid difference at or below the 20 CL positivity threshold (Figure 1 compared with Figure 2). Imputed SPM8 results support this finding, with a mild reduction of mean Centiloid difference for Vision∼Gemini from +5.6 CL to +4.9 CL and difference at 100 CL from +11.3 CL to +8.8 CL, but little change in differences for Vision∼mCT (Supplemental Figure 9 compared to Supplemental Figure 10).

To further demonstrate that PSF resolution alone does not account for measured differences between the Vision and Gemini, we show that 10 mm FWHM of smoothing of the Vision images is required to minimize the difference with Gemini native resolution Centiloid in Supplemental Material 1 (Supplemental Figure 2). Qualitatively, the 10 mm FWHM resolution Vision images had a much lower spatial resolution than the native Vision images (Supplemental Figure 3).

In contrast, Vision∼Gemini pairings did benefit from resolution harmonization for subjects with high Aβ levels, at 100 CL the difference reduce from +19.1 CL to +12.9 CL (Supplemental Figure 1^1^ and Supplemental Figure 1^2^). This benefit from resolution harmonization did not hold for subjects with low Aβ levels on the Vision∼Gemini, nor for any level of Aβ deposition on the Vision∼mCT.

The scanner mapping regressions fits for the Vision∼Gemini pair were calculated per AAL atlas region to investigate regional differences in scanner changes. The mapping slopes varied considerably, with regions in the frontal lobe as well as deeper structures (deep gray matter or gray matter in deep fissures, i.e. the post cingulate gyrus and insula) having a greater slope (Table 4). Regions in the temporal lobe were closest to a slope of unity. Supplemental Table 1 further depicts statistical differences between regions and shows that 55/66 (86%) of the possible region pairs are significantly different, whereas only 9/13 (69%) of the intra-lobular pairs are different, or 5/9 (55%) if the outlier regions in deep fissures, post cingulate gyrus and insular, are omitted. This indicates that the mapping changes over the brain but is more consistent locally. The mapping slopes were weakly associated positively with regional Aβ burden, but the relationship was not significant (Supplemental Figure 7). Full scatter plots of the mappings for Vision∼Gemini and Vision∼mCT are included in Supplemental Figures 6 and 5.

**Table 4.** Slope of regression fits for the Vision∼Gemini pair per subregion of the Centiloid mask.

Area	Region	Slope (CL/CL)
Frontal	Gyrus Rectus	1.08
	Orbitofrontal Cortex	1.14
	Dorsolateral Prefrontal Cortex	1.11
	Ventrolateral Prefrontal Cortex	1.12
Parietal	Post Cingulate Gyrus	1.10
	Superior/Inferior Parietal Lobules	1.06
	Angular	1.05
Temporal	Superior/Middle Temporal Gyri	1.04
	Inferior Temporal Gyrus	1.02
	Insula	1.12
Deeper Structures	Putamen	1.14
	Caudate Nuclei	1.13

Choice of reference region also impacted the scanner mapping with resolution harmonization (Table 5). The slope of the mapping between scanners, when comparing between reference regions, were all significantly different from one-another under a two-sided Mann-Whitney U rank test with Bonferroni correction for 6 tests. When the whole cerebellum and white matter were used as the reference region, the mapping slope was near-unity, measuring 2–3 CL less on the Vision than the Gemini (Supplemental Figure 6).

**Table 5.** Slope of regression fits for the Vision∼Gemini pair per reference region.

Reference Region	Slope (CL/CL)
Whole Cerebellum	1.10
Cerebral Gray Matter	1.14
Pons	1.06
Whole Cerebellum and White Matter	0.99

## Discussion

### Centiloid quantification was inconsistent across different scanners

Centiloid quantification on the Vision was significantly different from the Gemini and the mCT. In contrast, mCT and Gemini quantifications agreed more closely (see Supplemental Material 2), with a measurable statistically significant bias only occurring in low and borderline Aβ participants, and with a mean difference on the order of 1 CL. The relationships between scanners were found to be approximately linear, indicating that linear transform may be suitable to harmonize the measurements.

### Post-reconstruction resolution harmonization did not eliminate the scanner quantification differences

Resolution harmonization, using a smoothing kernel, was found to only marginally reduce (20% reduction in the best case, with the Gemini) the Centiloid difference between the Vision and other scanners. We further showed that a smoothing to 10–12 FWHM of the Vision would be required to minimize the difference with the Gemini; however, it was clear that the resulting images were not of the same resolution, further illustrating the inadequacy of the post-reconstruction resolution harmonization to account for the scanner differences. This suggests that the cause of discrepancy is not directly PSF resolution differences; although we speculate that the discrepancies, that are most different in the Vision, are due to the technological differences of digital PET's Lutetium-yttrium oxyorthosilicate crystals and silicone photomultiplier detector units. The differences between the mCT and Vision indicate that discrepancies are not directly due to the reconstruction algorithm or scatter correction algorithm, which are shared between the scanners. Therefore discrepancies may be due to interplay between resolution differences and the reconstruction or correction algorithms and their parameters, or other low-frequency confounding variables such as physical scatter effects.^38^ This is further supported by the results of the sub-regional analyses. If the Centiloid difference between scanners were primarily caused by high-frequency effects, the measured regression slopes in these analyses would have been consistent. Neighboring regions did indeed show similar regression slopes, however spatially distant regions exhibited quite different slopes, indicating low-frequency effects.

Joshi et al.^17^ previously investigated the impact of “high frequency correction” (which we term resolution harmonization) and “low frequency correction” in an ^18^F-FDG study, and found that resolution harmonization was able to reduce inter-scanner variability by 15%–25%. These results were consistent with the level of difference reduction at the 100 CL level found in this work, although overall is more than what we observed in our cohort. Joshi et al.^17^ found that correction for residual spatial effects did not further reduce inter-scanner variability. We did not investigate this correction; however, it is possible such a scheme would be more applicable to Aβ tracers or to the differences in modern scanners with higher resolution and sensitivity.

### Whole cerebellum with white matter as a reference region may aid harmonization for ^18^F-NAV4694

Brendel et al.^34^ previously demonstrated that the whole cerebellum with white matter reference region shows potential as a reference region that reduced longitudinal variation with ^18^F-Florbetapir. We have similarly found that this reference region resulted in a reduced inter-scanner variation. It is possible that part of the cause of the effectiveness of this reference region is the increased size and proximity to the Centiloid mask, increasing the correlation of low-frequency effects between the target and reference region. Alternatively, it may be that including the white matter in the reference region compensates, to a degree, for signal mixing of the target region with white matter due to resolution effects. While promising for harmonization, including white matter into the reference region may not be applicable to all Aβ tracers, and is not generally applicable to other PET tracers. Further, there may be concerns with including white matter in the reference region due to nonspecific binding being significantly different from the cortical gray matter.^39^ Therefore, this does not obviate the need for techniques to harmonize images acquired on different scanners.

### EM-PCA improved relationship estimation for undersampled scanner pairings

We also introduced a method using EM-PCA^40^ imputation to propagate information from other scanner pairs in order to better predict the relationship. We validated this technique with a leave-one-patient-out analysis (see Supplemental Material 2), demonstrating that propagating information from other scanner pairs to estimate missing data performs better than using only information from a given scanner pair. However, the relationships derived with this technique should be interpreted with care until the method can be further validated.

### PET-only analysis exhibits higher scanner Centiloid quantification bias, although benefits more from resolution harmonization

The PET-only CapAIBL quantification exhibited a higher scanner Centiloid quantification bias compared with the SPM8 analysis (Supplemental Figure 1^1^). This was expected, as the PET-only analysis relies on PET features for both spatial normalization and quantification. Unmatched resolution can lead to shrinking or expansion of the gray matter during non-rigid registration, and hence over- or under-estimation of the Centiloid mask. However, this also meant that resolution harmonization led to a higher level of improvement in inter-scanner quantification consistency (Supplemental Figure 1^2^). Resolution harmonization brought the inter-scanner bias closer to that of SPM8 (Figure 2); however, the SPM8 analyzed Centiloid measurements still exhibited more consistency.

### Limitations and future work

We have demonstrated the need for a harmonization algorithm able to correct for residual spatial differences between PET scanners, beyond resolution harmonization. We did not investigate the causal mechanisms, beyond demonstrating that the difference cannot be attributed to resolution effects and has a varying spatial effect. One of the plausible reasons for differences between different scanner models is the associated reconstruction software. Demonstrating exactly the effects would require an analysis of more scanners to compare inter- and intra-manufacturer scanner mapping variance.

This work only considered harmonization of resolution by post-reconstruction smoothing. A growing body of work proposes^41^ and investigates^42^ harmonization by selecting reconstruction parameters to minimize measured resolution between scanners as measured on phantom scans. The work presented here is equivalent to optimizing only the post-smoothing filter available on most systems, however, optimizing other parameters may further improve harmonization.

This work only contrasted a small subset of clinical PET scanners. However, there are a number of scanners available with much more different design and architecture, including dedicated brain scanners^43,44^ and large axial field-of-view scanners.^45,46^ It is possible such scanners also introduce differences beyond resolution to Centiloid quantification.

A subset of the dataset acquired on the Vision were also acquired with a reduced-dosage protocol (Table 2). Our dataset size was inadequately powered to distinguish an effect of dose from that of the Vision scanner, but it is possible that the dosage is affecting the measured scanner differences. However, this is unlikely given that previous analyses with various tracers all found that reducing dose to 50% resulted in much smaller effects than the differences we measure for the Vision.^47–50^ Further, all reports found that low dosage led to reduced apparent SUVr or Centiloid in AD individuals, whereas we identified an increased Centiloid measurement on the Vision.

In this work, we have presented a head-to-head scanner comparison, where scans were acquired within a year, whereas a more common time window for a head-to-head comparison would be 3 months. We have accounted for this with a correction using a population-based accumulation estimate and demonstrate the residual bias to be not significantly different from 0 in Supplemental Figure 1.

### Implications

Although quantitative Aβ burden assessment is not yet recommended in the clinic,^2^ it has been recommended for adoption,^51,52^ and improves reader accuracy against postmortem results.^53^ If and once adopted, the scanner differences will modify the eligibility of borderline patients for treatment. Scanner effects would also modify PET-based Aβ quantification during patient follow-up to inform dose regimen, as is done in the donanemab TRAILBLAZER-ALZ trials.^5^

It is likely patients may be impacted indirectly through biases and delays in clinical research. Inconsistent quantification between scanners introduces a confounding variable in multi-site, cross-sectional studies, where cohorts may be disproportionately scanned on different scanners due to site location demographics. In longitudinal studies, a scanner upgrade could amplify or reduce the measured effect of an intervention. Further, scanner differences introduce a marginal increase in measurement noise obscuring real A β changes.

### Conclusion

In this work, we have demonstrated that the type of PET Scanner that a participant is scanned on affects Centiloid quantification. We have further demonstrated that resolution harmonization does not correct for the observed scanner differences, and additionally that those differences are spatially-variant with a low spatial frequency over the Centiloid mask and reference-region-dependent. The effect was far less prominent between the Gemini and mCT pair, as compared to Vision paired with either of the aforementioned scanners. We conclude that harmonization for low-spatial-resolution effects may be required for some scanner pairings. Linear regression is adequate when paired data is available, as in this study, but image-based correction algorithms may be required in the absence of this data.

## Supplemental Material

sj-docx-1-alz-10.1177_13872877241313063 - Supplemental material for Digital detector PET/CT increases Centiloid measures of amyloid in Alzheimer's disease: A head-to-head comparison of cameras Supplemental material, sj-docx-1-alz-10.1177_13872877241313063 for Digital detector PET/CT increases Centiloid measures of amyloid in Alzheimer's disease: A head-to-head comparison of cameras by Ashley Gillman, Pierrick Bourgeat, Timothy Cox, Victor L Villemagne, Jurgen Fripp, Kun Huang, Rob Williams, Rosita Shishegar, Graeme O’Keefe, Shenpeng Li, Natasha Krishnadas, Azadeh Feizpour, Svetlana Bozinovski, Christopher C Rowe and Vincent Doré in Journal of Alzheimer's Disease
