# Supplementary material for: Digital detector PET/CT increases Centiloid measures of amyloid in Alzheimer's disease: A head-to-head comparison of cameras
Source: J Alzheimers Dis. 2025 Jan 26;103(4):1257–68. doi: 10.1177/13872877241313063 (PMC12231768; doi:10.1177/13872877241313063)
Supplement: sj-docx-1-alz-10.1177_13872877241313063 - Supplemental material for Digital detector PET/CT increases Centiloid measures of amyloid in Alzheimer's disease: A head-to-head comparison of cameras [file sj-docx-1-alz-10.1177_13872877241313063.docx]

# **Supplemental Material**

# **Digital detector PET/CT increases Centiloid measures of amyloid in Alzheimer's disease: A head-to-head comparison of cameras**

# **Supplemental Material 1**

# **Additional Methods and Figures**

## *Correction for scan delay*

Time between scans was limited to less than 365 days to reduce any impact of A$\beta$ accumulation. To correct any residual effects, population disease trajectory curves were used to calculate and correct for expected change in Centiloid between scans.^1^ Briefly, an independent population of 961 ^11^C-PiB and 45 ^18^F-NAV4694 scans from 236 AIBL subjects were quantified in Centiloid using each of the SPM8 and CapAIBL pipelines. Disease trajectory curves were created by fitting a cubic polynomial to the rates of change of Centiloid against mean Centiloid, and integrating the fitted curve to obtain a longitudinal trajectory of Centiloid against disease progression time (an arbitrarily-anchored time axis in units of years), repeated for each quantification pipeline, as previously described by Villemagne et al.^2^ and Burnham et al.^3^ Delay between scans was corrected by projecting Centiloid measurements to the time domain with the inverse curve, correcting for time between scans, and projecting back to corrected Centiloid using the curve.^3^ As no Vision scans were represented in the curve fitting dataset, Vision~Gemini and Vision~mCT pairs were corrected by transforming Gemini and mCT scan time, respectively, with the Vision scan as the reference time so that these measurements were unchanged. For Gemini~mCT pairs (Supplemental Material 2), the reference time was the midpoint between scans.

We validate the method by estimating the scanner mappings per the regression method outlined previously, each with and without applying scan delay correction, and then using the mapping to project all non-Vision scans to the Vision scanner. We plot the “scanner-adjusted” scan differences against the scan delay in Supplemental Figure 1, simulating a test-retest. Correcting for scan delay decreased both the mean and variance in difference between the scan pairs. The mean difference is much less than the test-retest variance both with and without correction.


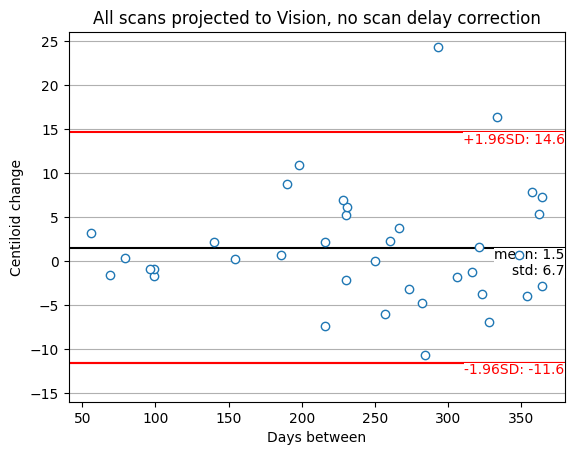

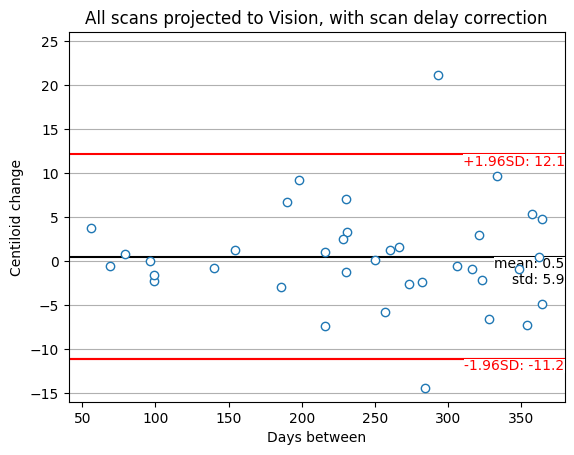


**Supplemental Figure 1.** Left) Centiloid temporal change between Vision scans, and other scans projected to Vision, by scan delay time. Right) The residual differences after scan delay correction.

## *Smoothing to minimize scanner differences*

We present an additional experiment here in which we increase the level of smoothing applied to the Vision scanner to find a value which minimizes the difference in Centiloid between the Vision and Gemini. Smoothing the Vision to 10 mm effective resolution is required to optimally match the Gemini (Supplemental Figure 2).


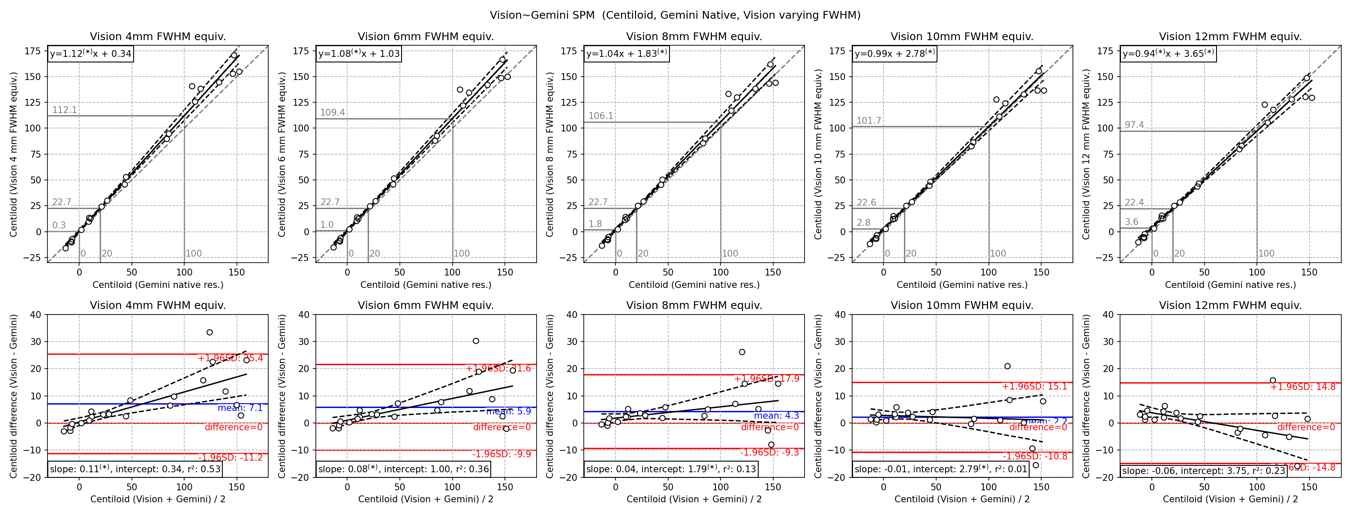


**Supplemental Figure 2.** Scatter and Bland-Altman plots for Vision and Gemini. All x-axes show Gemini Centiloid at native resolution. The first columns show Vision Centiloid at a matched resolution of 4, 6, 8, 10, and 12 mm equivalent, respectively.

Supplemental Figure 3 depicts matched Vision and Gemini scans smoothed to matched resolution (6 mm) and “forced harmonized”, with native resolution Gemini and 10 mm Vision. Although numerically harmonized in Supplemental Figure 2, qualitatively, the forced harmonized images do not appear similar.

|  |  | Vision | Gemini | **Supplemental Figure 3.** Representative patient scans acquired on Vision and Gemini (columns), with resolution harmonization and forces harmonization (see above) applied. Three patients per scanner are depicted, one each with low, borderline and high A$\beta$ burden. |
| --- | --- | --- | --- | --- |
| Low Amyloid | Harmonized  6mm | 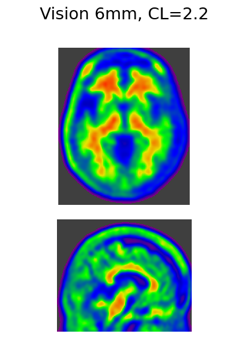 | 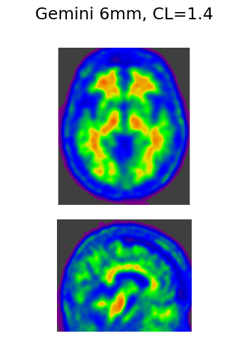 |  |
|  | Forced Harmonized 10mm | 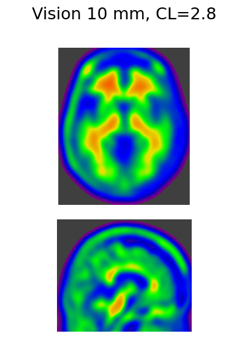 | 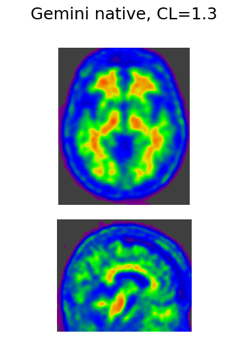 |  |
| Borderline Amyloid | Harmonized  6mm | 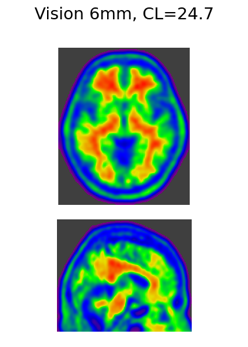 | 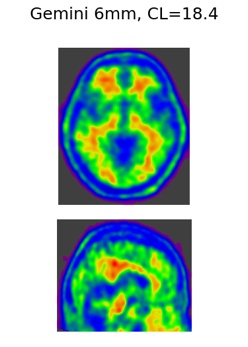 |  |
|  | Forced Harmonized 10mm | 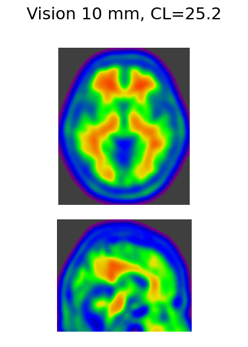 | 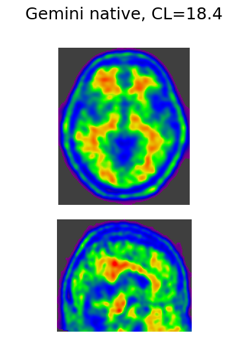 |  |
| High Amyloid | Harmonized  6mm | 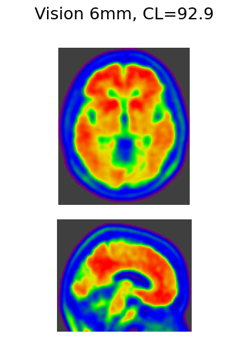 | 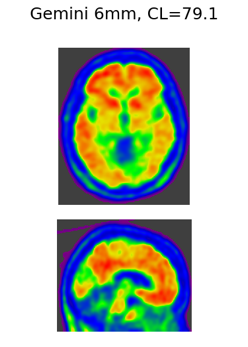 |  |
|  | Forced Harmonized 10mm | 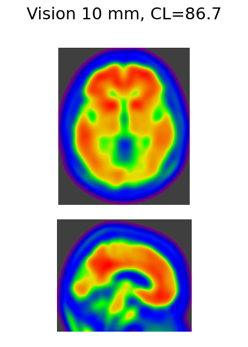 | 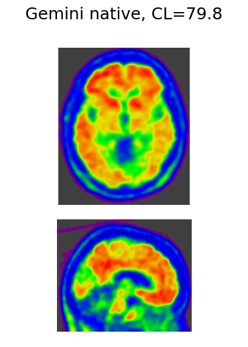 |  |

*Subregional analysis scatterplots*

The Vision~Gemini subregional analysis fit results, as summarized in Table 4, are shown in full in Supplemental Figure 4, and the results of statistical comparisons between all fits are given in Supplemental Table 1. The Vision~mCT subregional analysis fits are given in Supplemental Figure 5.


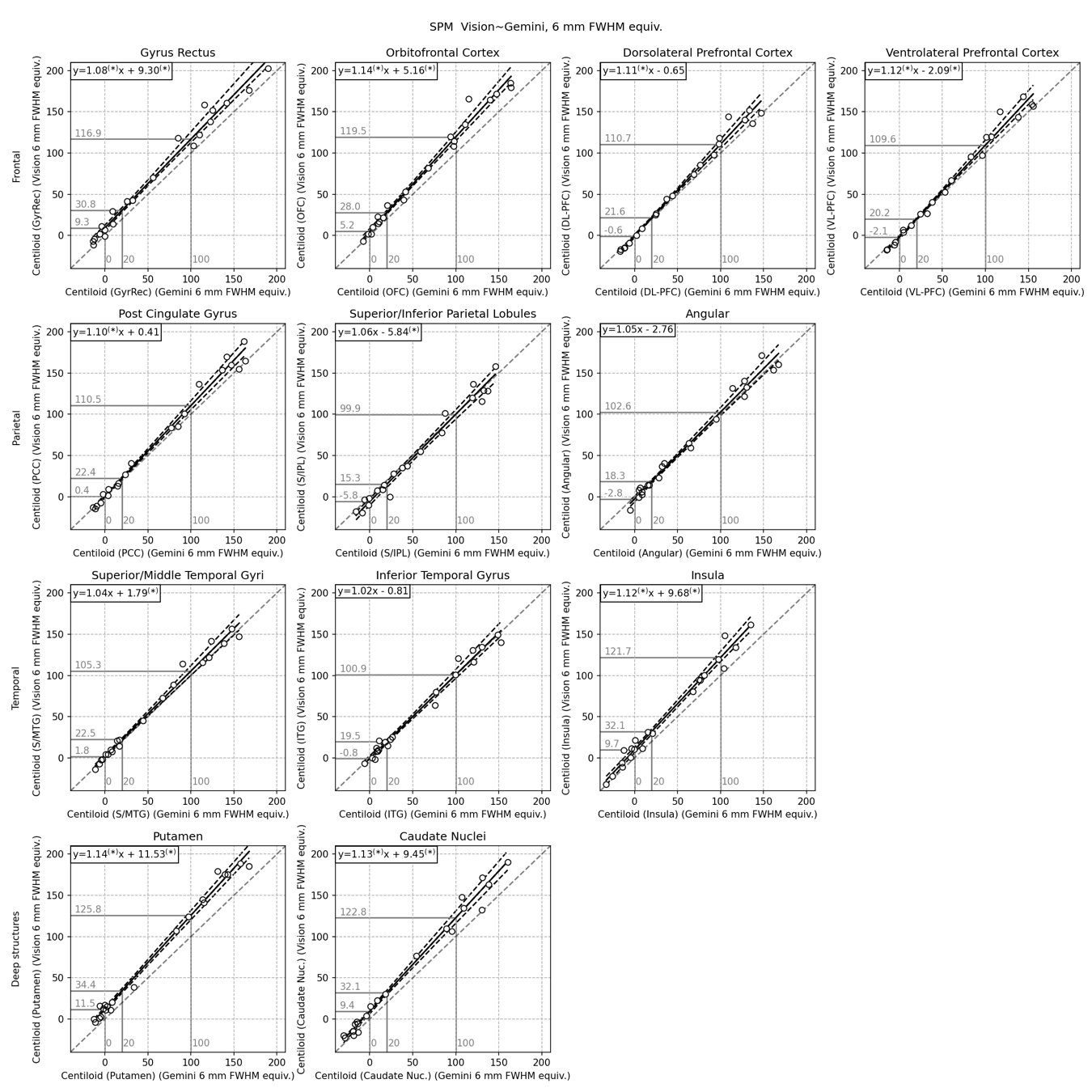


**Supplemental Figure 4.** Scatter plots of Vision~Gemini 6mm FWHM equivalent harmonized data, with a TLS regression line (black), equation (upper left) and the unity mapping (grey, dashed). Mappings are shown for 0, 20 and 100 Centiloid on the x-axis (grey). Each scatter plot depicts Centiloid of a different subregion of the Centiloid mask. Subregions are grouped by row, depicting subregions in the Frontal, Parietal, Temporal lobes and deep structures (physically, as opposed to any anatomical definition).

**Supplemental Table 1.** Calculated regression slopes per subregion of the Centiloid target mask for the Vision~Gemini scanner pair. Asterisks indicate significant differences (one for 5%, two for 0.5%) between the fit slopes between bootstrap estimates with a two-sided Mann-Whitney U rank test with Bonferroni correction for 66 tests. Region abbreviations in columns correspond to full region names in rows

|  |  |  | Frontal | | | | Parietal | | | Temp. | | | Deep Str. | |
| --- | --- | --- | --- | --- | --- | --- | --- | --- | --- | --- | --- | --- | --- | --- |
| Area | Region | **Slope (CL/CL)** | GR | OF | DL-PF | VL-PF | PCG | S/I-PL | Ang | S/M-TG | ITG | Ins | Put | CN |
| Frontal | Gyrus Rectus | 1.08 |  | ** | ** | ** | ** | ** | ** | ** | ** | ** | ** | ** |
|  | Orbitofrontal Cortex | 1.14 | ** |  | ** | ** | ** | ** | ** | ** | ** | ** |  | * |
|  | Dorsolateral Prefrontal Ctx. | 1.11 | ** | ** |  |  |  | ** | ** | ** | ** |  | ** |  |
|  | Ventrolateral Prefrontal Ctx. | 1.12 | ** | ** |  |  |  | ** | ** | ** | ** |  | ** | * |
| Parietal | Post Cingulate Gyrus | 1.10 | ** | ** |  |  |  | ** | ** | ** | ** | * | ** | ** |
|  | Sup./Inf. Parietal Lobules | 1.06 | ** | ** | ** | ** | ** |  |  | ** | ** | ** | ** | ** |
|  | Angular | 1.05 | ** | ** | ** | ** | ** |  |  | * | ** | ** | ** | ** |
| Temporal | Sup./Middle Temporal Gyri | 1.04 | ** | ** | ** | ** | ** | ** | * |  |  | ** | ** | ** |
|  | Inferior Temporal Gyrus | 1.02 | ** | ** | ** | ** | ** | ** | ** |  |  | ** | ** | ** |
|  | Insula | 1.12 | ** | ** |  |  | * | ** | ** | ** | ** |  | ** |  |
| Deep Structures | Putamen | 1.14 | ** |  | ** | ** | ** | ** | ** | ** | ** | ** |  |  |
|  | Caudate Nuclei | 1.13 | ** | * |  | * | ** | ** | ** | ** | ** |  |  |  |


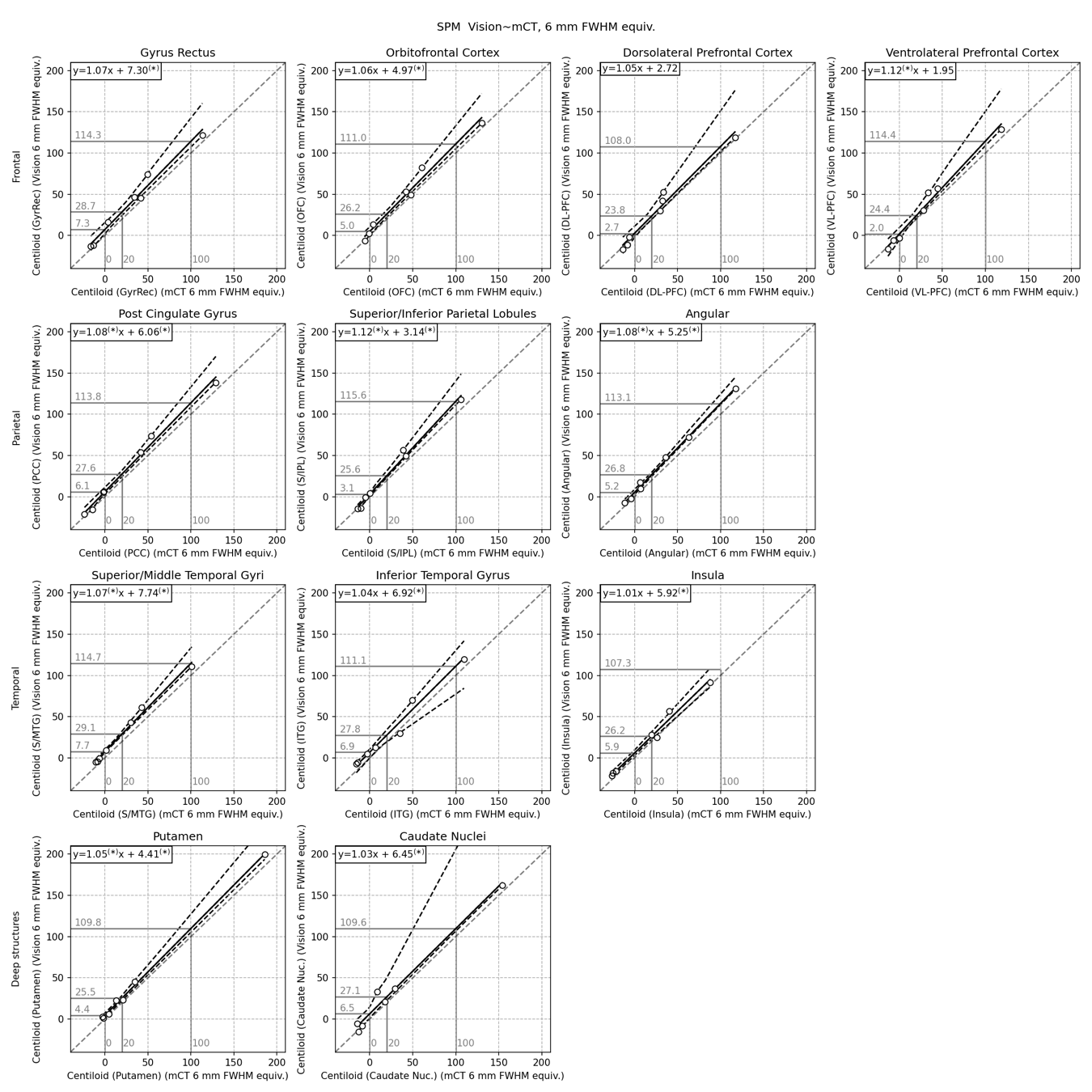


**Supplemental Figure 5.** Scatter plots of 6 mm FWHM equivalent harmonized data for Centiloid mask subregions, per Supplemental Figure 4 but for Vison~mCT.

The Vision~Gemini reference region analysis fit results, as summarized in Table 5, as well as those for Vision~mCT, are shown in full in Supplemental Figure 6.


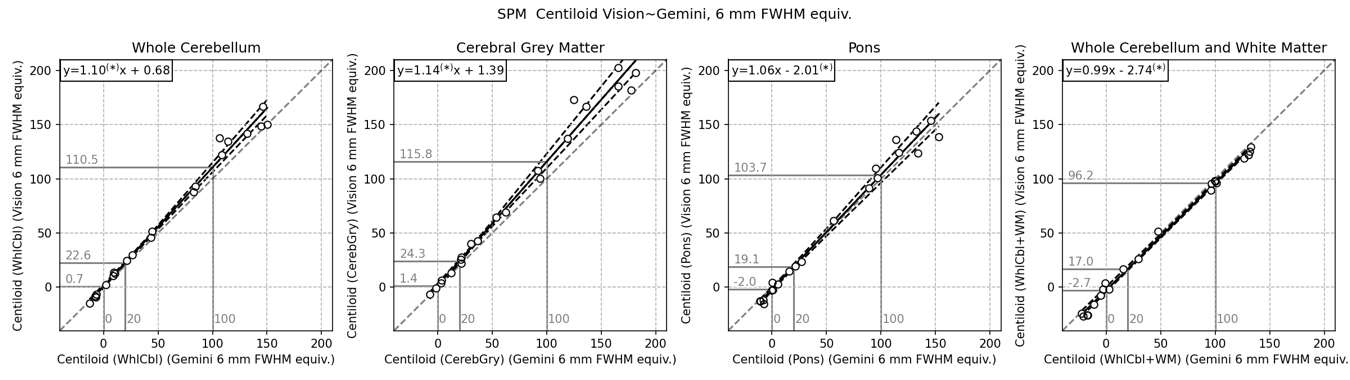

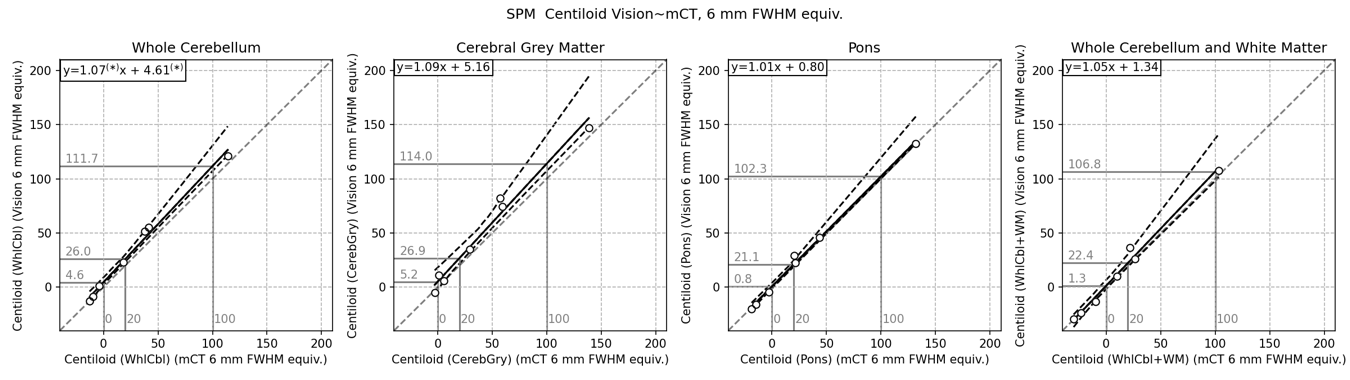


**Supplemental Figure 6.** Scatter plots of (Top) Vision~Gemini, (Middle) Vision~mCT and (Bottom) Gemini~mCT 6mm FWHM equivalent harmonized data, with a TLS regression line (black), equation (upper left) and the unity mapping (grey, dashed). Mappings are shown for 0, 20 and 100 Centiloid on the x-axis (grey). Each scatter plot depicts Centiloid calculated with a different reference region.

To determine whether the mapping slopes in Table 4 and Supplemental Table 1 are a function of the regional Aβ burden, we present the relationship between each region’s mapping slope (for the Vision~Gemini pair) and the mean regional Aβ burden in CL (mean over subjects and each scanner) for Aβ+ individuals (total target uptake > 20 CL) in Supplemental Figure 7. While there was a positive slope, the relationship was weak and non-significant.


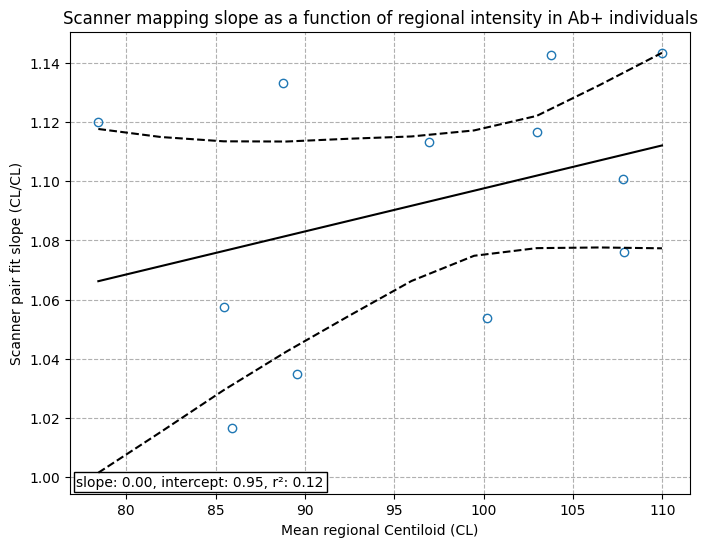


**Supplemental Figure 7.** Regional scanner mapping slope by regional Aβ burden of Aβ+ individuals.

Image difference analysis

Below we compare vowelized image differences with Centiloid differences between scanners. Image differences within the whole brain mask in SUVr (normalized to whole cerebellum) root-mean-square error (RMSE), and their relationship to Centiloid are presented in Supplemental Figure 8. Similar to Centiloid difference, the whole-brain RMSE increases with Centiloid.


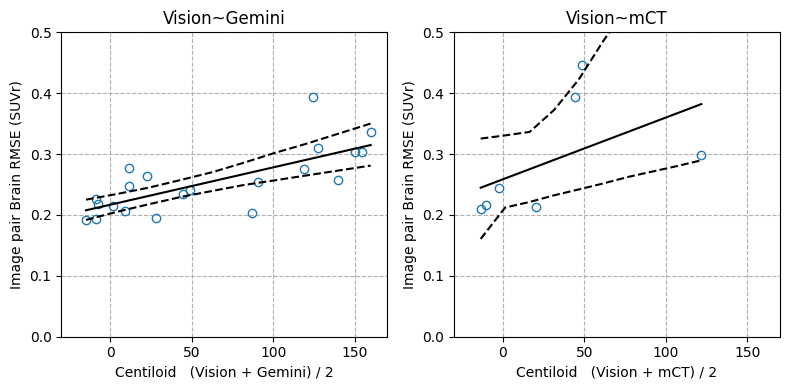


**Supplemental Figure 8.** Root-mean-square error of paired images at native resolution in cerebellum SUVr, against mean Centiloid, for each scanner pair.

# **Supplemental Material 2**

# *Analysis with imputation*

The participant recruitment on the mCT scanner was low, especially for high CL participants, meaning it was difficult to draw conclusions about the relationships with this scanner. Given a scanner pairing A∼B, information from a pairing with another scanner pair, A∼C can be informative if the relationship B∼C is also known. That is, A∼C can be transformed into A∼B by mapping the C measurements to B via the B∼C relationship, and incorporated with already-known A∼B information. Similarly, B∼C can be transformed into A∼B via a known A∼C.

In this section, we treat this problem as a missing data problem, in which missing scanner Centiloid measurements for each paired scan are imputed. This is achieved, in an unbiased manner (that is, without any preferred scanner: the result is independent of the scanner order) using expectation-maximization principal component analysis (EM-PCA).^4^ EM-PCA was chosen for imputation at PCA is a natural generalization to *n*-dimensions of the TLS regression performed in the regression analysis, so can be understood as performing the regression analysis with all scanners simultaneously. This is an iterative algorithm, in which PCA is iteratively performed to estimate new values for the missing data via a projection to a low dimensional (in this case, 1 dimensional) manifold using all data points, including the missing ones, from the previous iteration. The missing data are initially set to the dataset mean.

To validate, we performed a leave-one-patient-out analysis, for each patient estimating their Centiloid burden measured on one scanner based on the measurement of their other scan on the second scanner, performed in both directions. For reference, naively regressing all scanner pairs with TLS and propagating to all scanner pairs, each patient was able to be predicted with an error of 8.8 CL root-of-mean-square-error (RMSE). Using the proposed EM-PCA imputation method, each patient was able to be predicted with an error of 7.7 CL RMSE.

Further, using only the Vision∼Gemini data and TLS regression, each patient was able to be predicted with an error of 10.7 CL RMSE. This indicates that including patients from the mCT scanner improves the estimate of the relationship between even the highest-sampled pairing, the Vision∼Gemini pair, and suggests that it is likely to further help the lower-sampled pairs with the mCT.

An independent set of analyses were conducted using a dataset where the “missing scanner” from each scanner pair was imputed. This allowed information from scanner pairs with less acquired data to be approximated from those with more data acquired. Supplemental Figure 9 shows the native resolution scatter and Bland-Altman plots, and the corresponding 6 mm resolution harmonized results are depicted in Supplemental Figure 10. These plots correspond to Figures 1 and 2, with the imputed data included.


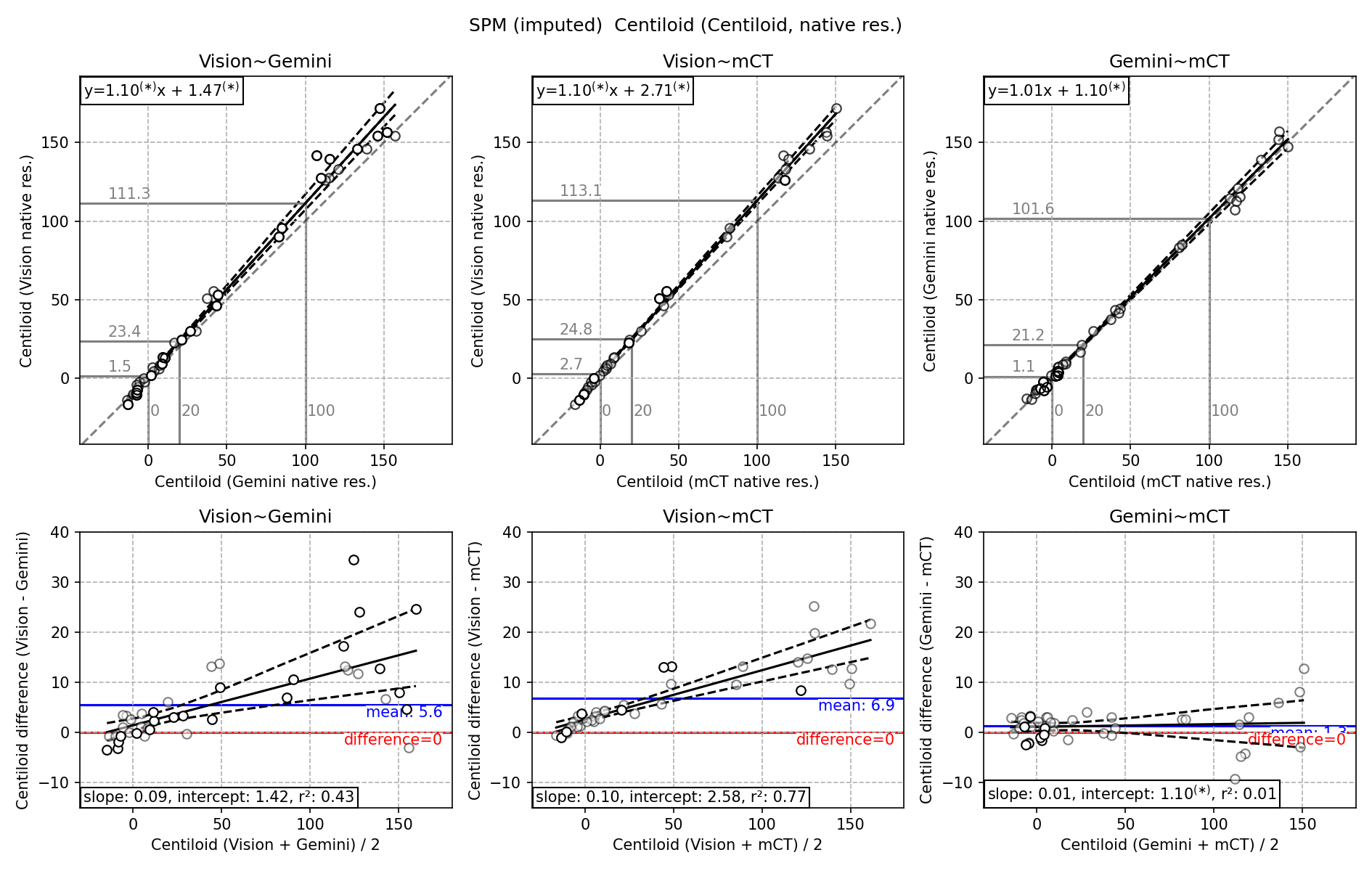


**Supplemental Figure 9.** Scatter and Bland-Altman plots at native resolution, per Figure 1, with EM-PCA imputation. Circles with solid contours represent real measurements, while semi-transparent circles represent imputed measures.

The imputed analysis of head-to-head data was able to narrow the bootstrap confidence interval for the Vision∼mCT pair and generate a mapping estimate for the Gemini∼mCT pair. The following observations become clearer in Supplemental Figure 9. Firstly, the Gemini and mCT quantify Centiloid similarly. The Gemini exhibits a near-constant difference of +1 CL compared to the mCT. Secondly, as was apparent before imputation, the quantification of Centiloid on the Vision is significantly different from the mCT and Gemini and further exhibits an error that correlated with Centiloid. Thirdly, the variance in the Centiloid quantification mapping also correlates with Centiloid. This is likely not a function of the scanners, and rather indicative that the test-retest error of Centiloid quantification is higher for high Centiloid individuals. However, it is noteworthy that the variance is significantly lower, and the coefficient of determination (*r*^2^), higher, for the Vision∼mCT pairing than the Vision∼Gemini. This could suggest that the variance is a function of the scanner change, and that the fact that the Vision and mCT are both Siemens scanners and share a reconstruction and scatter estimation algorithm which improves the quantification consistency.

The use of imputation in the dataset did not change the conclusion that resolution harmonization fails to correct for scanner differences (Supplemental Figure 10). The Vision pairings continue to exhibit a Centiloid-correlated bias with a slight reduction due to resolution harmonization, and the Gemini∼mCT pairing bias similarly only was slightly reduced.


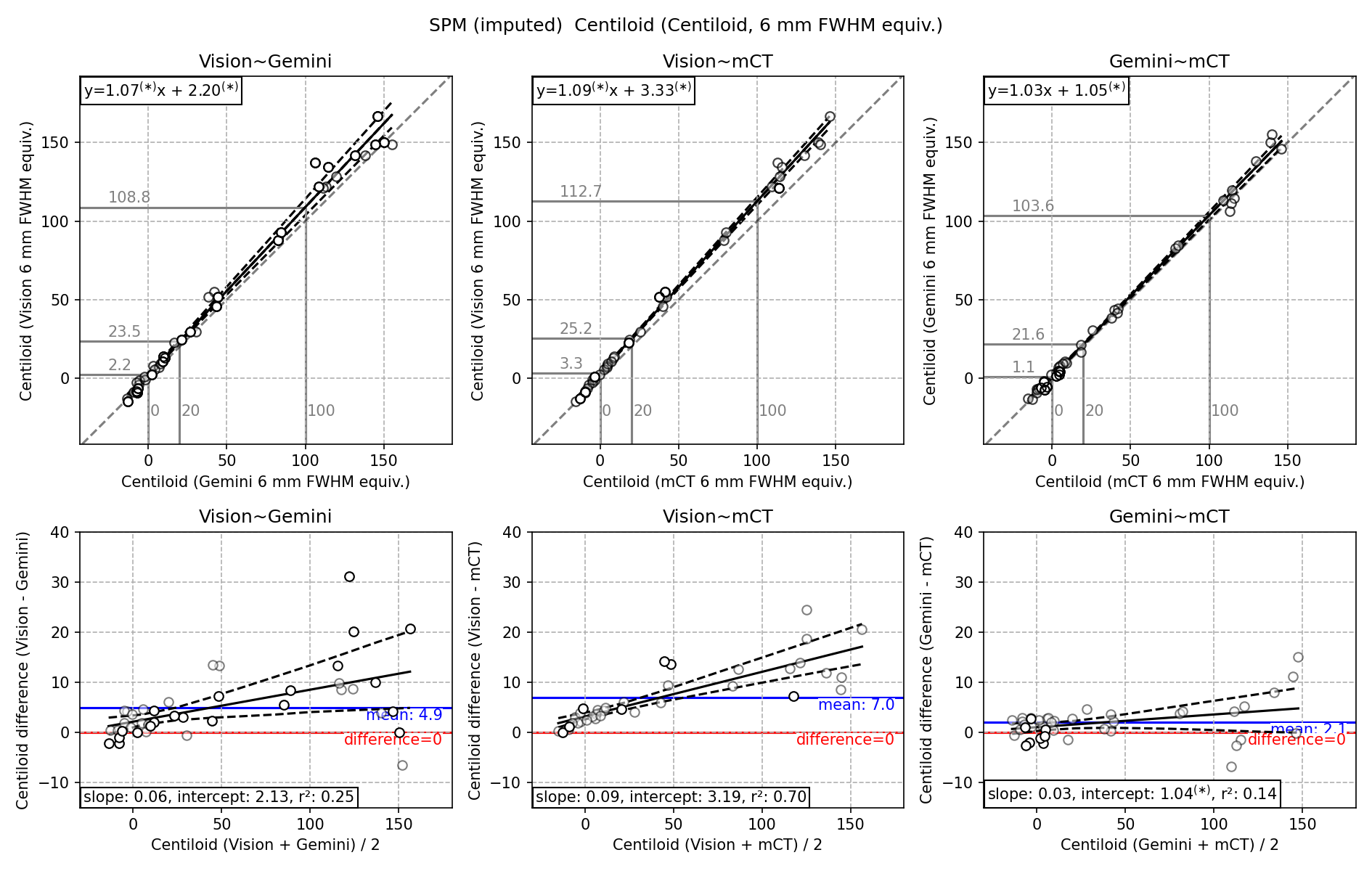


**Supplemental Figure 10.** Scatter and Bland-Altman plots at 6 mm harmonized resolution, per Figure 2, with EM-PCA imputation. Circles with solid contours represent real measurements, while semi-transparent circles represent imputed measures.

# **Supplemental Material 3**

# *Analysis with CapAIBL (PET-only)*

In the CapAIBL processing, the PET images were registered to an adaptive template, optimized to match the pattern of Aβ retention.^5^ The optimal template was then used as a target for the non-rigid registration for spatial normalization. After spatial normalization, the standardized uptake value ratio (SUVr) was calculated using the ratio of mean activity in the Centiloid neocortical mask^6^ to that in the chosen reference mask, which unless otherwise specified was the whole cerebellum. Otherwise, CapAIBL analyses were performed identically to those of SPM8.

Supplemental Figure 11 shows the native resolution scatter and Bland-Altman plots, and the corresponding 6mm resolution harmonized results are depicted in Supplemental Figure 12. These plots correspond to Figures 1 and 2, with PET-only analysis (no MR-based spatial normalization) using CapAIBL.

The PET-only CapAIBL quantification exhibited a higher scanner Centiloid quantification bias compared with the SPM8 analysis (Supplemental Figure 11). This was expected, as the PET-only analysis relies on PET features for both spatial normalization and quantification. Unmatched resolution can lead to shrinking or expansion of the grey matter during non-rigid registration, and hence over- or under-estimation of the Centiloid mask. However, this also meant that resolution harmonization lead to a more significant improvement in inter-scanner quantification consistency (Supplemental Figure 12). Resolution harmonization brought the inter-scanner bias closer to that of SPM8 (Figure 2); however, SPM8 still exhibited a superior performance in this regard.


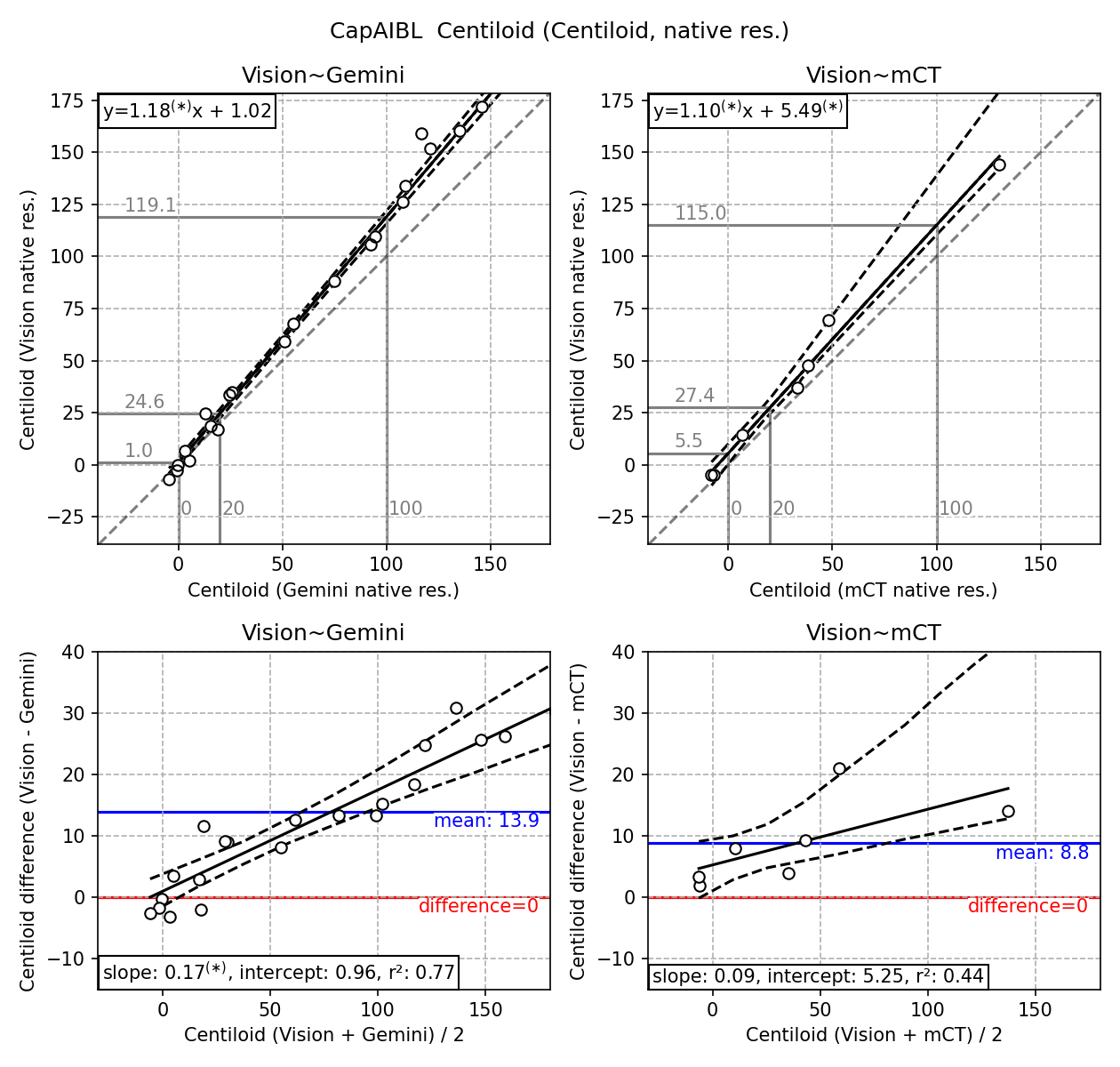


**Supplemental Figure 11.** Scatter and Bland-Altman plots at native resolution, per Figure 1, with CapAIBL Centiloid quantification.


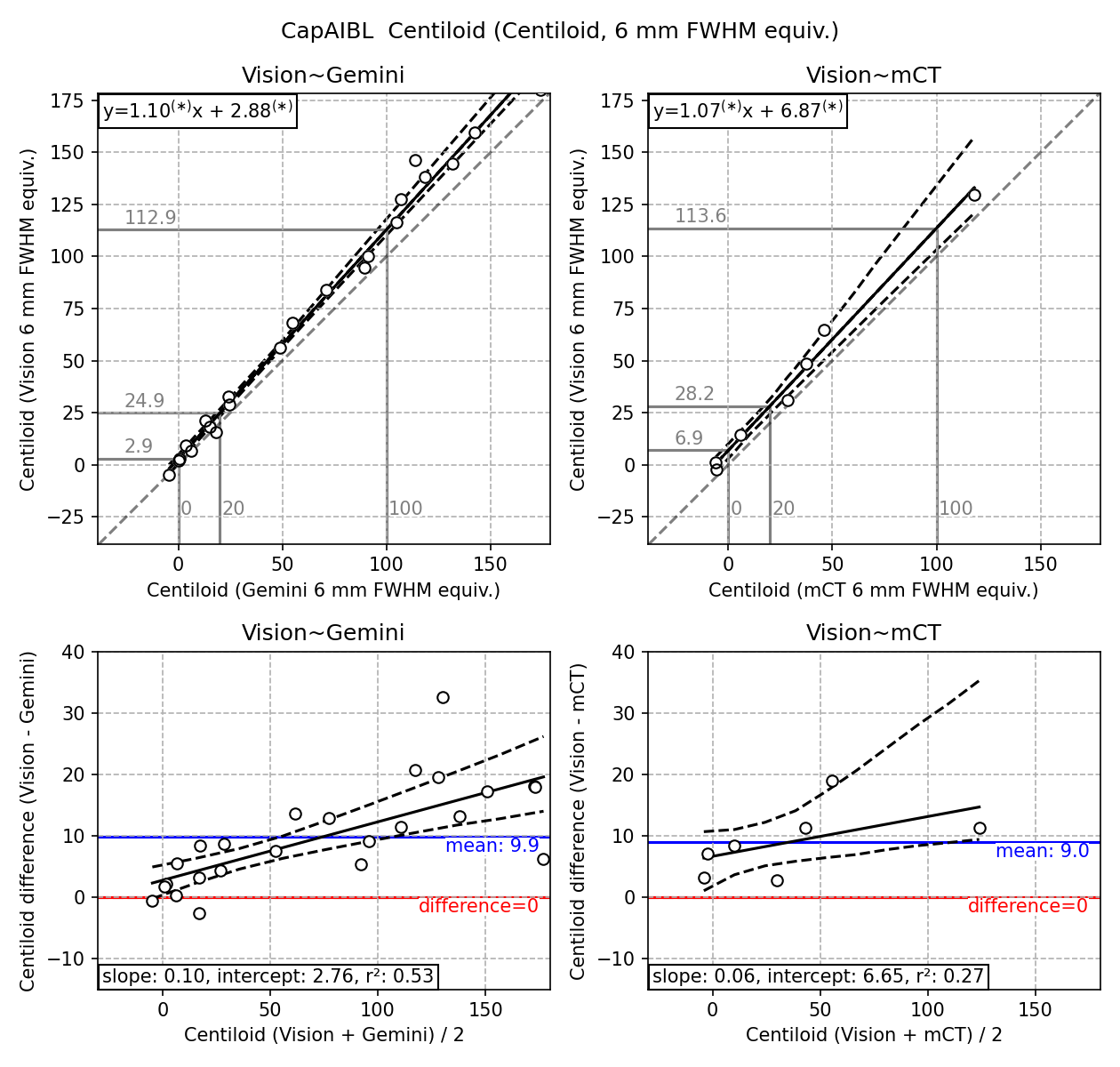


**Supplemental Figure 12.** Scatter and Bland-Altman plots at 6 mm harmonized resolution per Figure 2, with CapAIBL Centiloid quantification.

# **References**

1. Cox T, Bourgeat P, Dore V, et al. Comparing the longitudinal progression of CSF biomarkers with PET Amyloid biomarkers for Alzheimer’s disease. *Alzheimers Dement* 2022; 18: e068082.

2. Villemagne VL, Burnham S, Bourgeat P, et al. Amyloid β deposition, neurodegeneration, and cognitive decline in sporadic Alzheimer’s disease: a prospective cohort study. *Lancet Neurol* 2013; 12: 357–367.

3. Burnham SC, Cox T, Benzinger T, et al. When does Alzheimer’s disease start? Robust estimates based on longitudinal Aβ-amyloid-PET in three large international cohorts. SSRN 2024; DOI: 10.2139/ssrn.4757682 [Preprint]. Posted 18 March 2024

4. Roweis S. EM algorithms for PCA and SPCA. In: *Advances in Neural Information Processing Systems*. Cambridge, MA: MIT Press, https://proceedings.neurips.cc/paper_files/paper/1997/hash/d9731321ef4e063ebbee79298fa36f56-Abstract.html (1997, accessed 31 May 2023).

5. Bourgeat P, Villemagne VL, Dore V, et al. Comparison of MR-less PiB SUVR quantification methods. *Neurobiol Aging* 2015; 36: S159–S166.

6. Klunk WE, Koeppe RA, Price JC, et al. The Centiloid Project: Standardizing quantitative amyloid plaque estimation by PET. *Alzheimers Dement* 2015; 11: 1-15.e4.
